# Training emotion recognition in depression—An experimental study

**DOI:** 10.1111/bjc.12540

**Published:** 2025-03-18

**Authors:** Mira A. Preis, Katja Schlegel, Sophie Rehbein, Katja Lorenz, Timo Brockmeyer

**Affiliations:** ^1^ Department of Clinical Psychology and Psychotherapy University of Goettingen Göttingen Germany; ^2^ Institute of Psychology University of Bern Bern Switzerland; ^3^ Clinical Psychology and Translational Psychotherapy Unit, Institute of Psychology University of Münster Münster Germany

**Keywords:** depression, emotion perception, emotion recognition, social cognition, theory of mind

## Abstract

**Background:**

Patients with depression often show a reduced emotion recognition ability (ERA), which is considered to contribute to interpersonal difficulties and thereby to the development and maintenance of the disorder. In light of the lack of experimental studies testing this hypothesis, the present study investigated whether a single session of computerized training can enhance ERA in patients with depression and whether this affects interpersonal problems and symptoms of depression.

**Methods:**

Forty outpatients with major depressive disorder or persistent depressive disorder were randomly assigned to a single session of either computerized training of ERA (TERA) or a sham training. One day prior to and 14 days after training, ERA, interpersonal problems and symptoms of depression were recorded.

**Results:**

Both groups showed significant improvements in ERA and in symptoms of depression. Participants who received TERA showed greater improvements in ERA than participants who received sham training. However, the groups did not differ regarding changes in symptoms of depression, and none of the groups showed significant changes in interpersonal problems.

**Conclusions:**

A single session of computerized training can effectively improve ERA in patients with depression. In the short term, however, TERA neither affected interpersonal problems nor symptoms of depression.

**Practitioner Points**

- This is a pilot randomized controlled trial with 40 patients with depression.
- Single‐session computerized training of emotion recognition ability (TERA) was tested against sham training.
- TERA improved emotion recognition ability in patients with depression but not in depressive symptoms.

## INTRODUCTION

With an estimated lifetime prevalence of approximately 20% (Hasin et al., 2018), major depressive disorder (MDD) constitutes one of the most prevalent, chronic and debilitating mental disorders as well as one of the leading causes of years lived with disability (James et al., 2018; Krause et al., 2021). Although treatment options for MDD, especially cognitive behaviour therapy (CBT) and medication, are effective (Cuijpers et al., 2020), response rates of 41–66.5% indicate that there is a substantial group of patients who do not benefit from existing treatments (Cuijpers et al., 2021; Kelley et al., 2018). Furthermore, meta‐analyses have shown that about one‐third of patients, after being treated with CBT or medication, experience relapse or recurrence after no more than 3 years (Gueorguieva et al., 2017). Thus, there is an urgent need to better understand processes involved in the maintenance and recurrence of depression, and to develop novel treatments.

In addition to the significant economic burden for society (Greenberg et al., 2015), MDD affects patients and their caregivers in various ways (Balkaran et al., 2021; Kamenov et al., 2016). Kamenov et al. (2016) identified interpersonal activities as one of the most affected functioning domains in patients with depression, which had a strong impact on the quality of life in these individuals. Correspondingly, lack of social support and interpersonal stress are associated with MDD onset and a poor prognosis (Buckman et al., 2021; Santini et al., 2015; Vrshek‐Schallhorn et al., 2015). Of particular relevance to the present study is that difficulties in the interpersonal domain are partly attributed to reduced emotion recognition ability (ERA) in patients with depression (Krause et al., 2021).

Identifying and interpreting others' emotional states is crucial for social interaction and interpersonal relationships (Bourke et al., 2010; Schlegel, Fontaine, & Scherer, 2017). In healthy individuals, higher ERA is associated with better interpersonal functioning and higher well‐being, whereas low ERA is linked to difficulties in social interaction, frustration and negative affect (e.g. Elfenbein et al., 2007; Hall et al., 2009; Schlegel et al., 2018; Wang et al., 2019).

Meta‐analyses have shown that MDD is associated with reduced ERA (Dalili et al., 2015; Krause et al., 2021). In addition, there is evidence that poor ERA predicts MDD onset and recurrence (Vrijen et al., 2016; Yamada et al., 2015). Krause et al. (2021) have pointed out the potential reciprocity of the relationship between reduced ERA and depression. Neuroanatomical and neurochemical changes in MDD might interfere with emotional processing, which in turn might impair social functioning and thereby sustain depression. In addition, individuals with reduced ERA might be more vulnerable to interpersonal problems and thereby to develop depression (Krause et al., 2021; Kupferberg & Hasler, 2023).

However, via different interventions, ERA can be trained in healthy subjects (Schlegel, 2021; Schlegel, Vicaria, et al., 2017), in volunteers with elevated symptoms of depression (Penton‐Voak et al., 2021) and in patients with other mental disorders such as autism spectrum disorder (Berggren et al., 2018). To this end, Schlegel et al. (2018) developed a computerized ERA training (TERA) that teaches participants to differentiate 14 emotions. TERA has been proven to successfully enhance ERA both in healthy individuals (Schlegel, 2021; Schlegel et al., 2018) and in patients with anorexia nervosa (Preis et al., 2020). In the latter study, patients who received TERA showed not only improved ERA but also short‐term reductions in eating disorder symptoms (Preis et al., 2020).

Following the experimental psychopathology approach (Field et al., 2019) and in line with one of our previous studies on ERA in patients with anorexia nervosa (Preis et al., 2020), our aim in the present study was to investigate the extent to which strengthening ERA affects depressive symptoms. We hypothesized that TERA in patients with depression would lead to greater improvements in ERA, interpersonal problems and depressive symptoms than a sham training.

In a recent study, no effect of an ERA training on depressive symptoms was found (Penton‐Voak et al., 2021). However, this study differed from the current study in important ways. Penton‐Voak et al. (2021) used an interpretation bias modification training in which participants were trained to perceive happiness over sadness in ambiguous emotional expressions while we used a training in which participants were taught to correctly interpret various nonverbal cues of a broad range of emotions. In addition, the Penton‐Voak et al.'s (2021) study examined volunteers with elevated depression scores, whereas we examined a clinical sample of patients with diagnosed depression. Since a recent meta‐analysis (Krause et al., 2021) showed that mainly patients with more severe depression exhibit reduced ERA, it could be that participants in the Penton‐Voak et al. (2021) study had rather mild difficulties in emotion recognition, which may not have had a strong impact on their (rather mild) depressive symptoms.

## METHODS

### Study design

In this experimental study, outpatients with depression were randomly assigned to receive a single session of either TERA or a sham training (control group). Changes in ERA served as the primary outcome and changes in depressive symptoms and interpersonal problems as secondary outcomes. Outcomes were assessed at baseline and 2 weeks after training. Study approval was obtained by the local ethics committee of the Institute of Psychology at the University of Goettingen. Data, analytic methods and study materials are available from the corresponding author upon reasonable request.

### Sample

Previous research revealed large effect sizes for the superiority of TERA over sham training with regard to ERA improvements in healthy individuals (Schlegel et al., 2018). Since TERA has not been previously examined in patients with depression, we chose a more conservative approach and expected only a medium effect size. An a priori power calculation indicated that a total sample size of *n* = 34 would be needed in order to detect a medium effect size in a 2 × 2 mixed ANOVA with two groups (80% power, .05 two‐tailed significance level). A data loss of up to 10% due to technical errors and dropouts was assumed (i.e. *a* = .1). Applying an attrition correction factor of 1/(1−*a*) resulted in a sample size of *n* = 38, which was rounded up to *n* = 40.

Participants were recruited from the waiting list of an outpatient clinic. Participants had to be ≥18 years old, meet DSM‐5 criteria of MDD or persistent depressive disorder (PDD) (American Psychiatric Association, 2013) and were only deemed eligible if they had not received more than five diagnostic/treatment sessions yet at the outpatient clinic. Study assessors (master‐level students in clinical psychology) screened participants for DSM‐5 criteria of MDD/PDD using the corresponding modules of the Structured Clinical Interview for DSM Disorders (SCID; First et al., 2016). Exclusion criteria were comorbid psychosis, borderline personality disorder, bipolar disorder, substance use disorder or insufficient knowledge of the German language to complete the tasks. Participants received financial compensation (€40) for their participation. All participants provided written informed consent.

Two participants withdrew their consent (one after study completion, one after pre‐assessment), five participants dropped out (12.5%; four participants before treatment, one after treatment) and the data of one participant were lost due to technical errors. Thus, the treatment completer sample comprised 16 participants in the TERA group and 17 in the control group (i.e. *N* = 33 in total).

### Measures

#### Emotion recognition ability (ERA)

The short version of the Geneva Emotion Recognition Test (GERT‐S; Schlegel & Scherer, 2016) was used to measure ERA. This computer‐based test includes 42 short video clips (each 1–3 s) borrowed from the Geneva Multimodal Emotion Portrayals database (GEMEP; Bänziger et al., 2012) that display 14 different emotions (pride, joy, amusement, pleasure, interest, relief, anger, fear, despair, irritation, anxiety, sadness, disgust, surprise). Each clip shows one out of 10 actors (5 women, 5 men; younger, middle‐aged and older individuals) from their upper torso upwards expressing one of the described emotions while saying a pseudo‐linguistic sentence (a sentence without semantic meaning). All clips contain multimodal nonverbal cues (i.e. prosody, facial expression, gestures, posture), adding to the ecological validity of the task. After each clip, participants rate which of the 14 emotions they think the actor portrayed. The participants' answers are coded as correct (1) or incorrect (0), resulting in a total average GERT‐S score between zero and one. Schlegel and Scherer (2016) have demonstrated good internal consistency and construct validity of the GERT‐S. Importantly, the GERT‐S comprises only stimuli that are not part of the training.

#### Symptoms of depression

The PHQ‐9 (Kroenke et al., 2001) is a commonly used instrument to assess depression severity. Regarding the last 2 weeks, participants rate their experience of each of the nine DSM criteria for MDD on a scale from 0 (not at all) to 3 (nearly every day). Total scores can range between 0 and 27, with higher scores representing greater symptom severity. Kroenke et al. (2001) have demonstrated its reliability and validity.

#### Interpersonal problems

Interpersonal problems were assessed by a short version (IIP‐12; Lutz, 2002; Lutz et al., 2006) of the Inventory of Interpersonal Problems (IIP; Horowitz et al., 1988). Based on the circumplex model of interpersonal behaviour, participants rate 12 items on a 5‐point Likert scale ranging from 0 (not) to 4 (very). Following Lutz (2002), the global sum score was used, which showed acceptable to good internal reliability in previous studies (Lutz, 2002).

### Interventions

#### TERA

TERA consists of a single session of computerized, audiovisual training (approx. 35 min) which includes an instruction part and a practice‐with‐feedback part, both teaching participants to recognize and differentiate among 14 different emotions (Schlegel et al., 2018). The instruction part consists of written descriptions and video clips in which an experimenter explains the meaning of and nonverbal cues related to each emotion. Each explanation is followed by two example video clips (each 1–3 s) from the GEMEP to illustrate these cues (Bänziger et al., 2012). As described above for the GERT‐S (Schlegel & Scherer, 2016), each clip conveys various nonverbal (i.e. facial, gestural, postural and auditory) emotional cues. Participants can replay the example clips up to three times and reread the description of the nonverbal cues while watching. In the practice‐with‐feedback part, 42 additional GEMEP video clips are presented. After each clip, participants have to decide which of the 14 emotions was expressed by the actor and receive immediate feedback about whether their answer was correct or incorrect. Correct responses are followed by the next clip. In the case of an incorrect answer, the video is replayed and the participant can again identify which emotion it was. If the second answer is still wrong, the correct emotion is displayed and the participant proceeds to the next clip.

#### Sham training (control group)

The sham training (Schlegel et al., 2018) was also delivered in a computerized, audiovisual fashion. However, participants in this group did not learn to categorize emotions but 14 different types of clouds. In accordance with TERA, the characteristic features (e.g. altitude, opaqueness, shape) of each cloud type, such as Cirrus or Nimbostratus, were described by an experimenter in a video clip followed by a written description and two pictures of each cloud type. The practice‐with‐feedback part included 42 pictures of clouds that had to be categorized by the participants. Instructions and feedback were given according to the same structure as in the real training.

### Procedure

After an initial screening interview via telephone, participants attended three online appointments (pre‐assessment, training session, post‐assessment). At pre‐assessment, we assessed ERA, depressive symptoms and interpersonal problems. Afterwards, participants were randomly assigned to one of the two groups (TERA/control group). Randomization was performed in a 1:1 ratio without stratification, using an online tool (www.randomizer.org). On the next day, participants were sent a link to the online training (TERA/cloud training) via e‐mail. Two weeks after participants had finished the training, ERA, depressive symptoms and interpersonal problems were reassessed. Latencies to finish the post‐assessment after receiving the invitation mail varied between subjects. Some participants completed the tasks almost immediately, whereas others needed multiple reminders. However, these latencies did not differ significantly between groups (see Results section).

### Statistical analysis

All statistical analyses were carried out using IBM SPSS Statistics version 25 (SPSS Inc., IBM Corporation, Armonk, NY, USA). Data quality and completeness were determined by graphical methods and descriptive statistical analyses. Since this is not considered a clinical trial examining the efficacy of a treatment but an experimental proof‐of‐concept study examining the potential causal role of ERA on depressive symptoms, we chose to conduct a per protocol analysis, that is, only participants were included who actually carried out the training and took part in post‐assessment. As no participant's scores deviated more than 3.29 SD from the group's mean, all analyses were based upon the treatment completer sample of *n* = 33 (*n* = 16 TERA, *n* = 17 sham). Statistical tests were performed two‐tailed, with the significance value set to *p* < .05.

Potential group differences at pre‐assessment were examined using *t*‐tests for independent samples. Changes in outcomes were assessed by means of 2 × 2 mixed ANOVAs with group (TERA vs. control) as a between‐subjects factor and time (pre‐ vs. post‐training) as a within‐subjects factor. The calculation of the effect size for the group × time interaction effects in the repeated measures design follows the recommendations of Morris and DeShon (2002), according to which pretest differences and sample sizes are taken into account and the differences in the pre‐post measurement in the groups are weighted by the pooled standard deviation of the pre‐measurement, as this was not influenced by the intervention. In addition, we report within‐group effect sizes for the magnitude of change in outcome variables, separately for each treatment condition.

Exploratory moderator analyses using all available baseline variables (without necessary reference to earlier empirical findings or theoretical models) were performed using PROCESS v4.0 (Hayes, 2017) to examine whether age, gender, number of previous psychotherapy sessions in the outpatient clinic in the recent past, medication, depressive symptoms at pre‐assessment or time (days) between training and post‐assessment influenced the effects of treatment condition (TERA/control) on changes in outcomes. These analyses were Bonferroni‐adjusted due to multiple testing (6 moderator variables; therefore *p* < .008). Bootstrapping with 5000 re‐samples was employed to compute (Bonferroni‐corrected) 99% bias‐corrected and accelerated confidence intervals.

## RESULTS

### Sample characteristics at pre‐assessment

The treatment completer sample (*n* = 33) was 36.48 ± 13.08 years old on average. At pre‐assessment, groups did not differ significantly in any of the variables except gender (less women in the TERA group; see Table 1). However, there were no gender differences in ERA, depressive symptoms or interpersonal problems at baseline (all *p* > .42). Groups did also not differ regarding the proportion of patients with major depression versus persistent depressive disorder. Furthermore, groups did not differ concerning the average time span between training and post‐assessment (*M*_TERA_ = 19.00 days, *SD*_TERA_ = 6.51, *M*_Control_ = 22.88 days, *SD*_Control_ = 9.75, *t*(31) = −1.34, *p* = .19, *d* = .47). Even if there are some medium effect sizes for group differences at baseline, these are considered irrelevant for the further analyses due to the repeated measures design and applicable conventions for randomized controlled trials (Moher et al., 2010).

**TABLE 1:** Sample characteristics at pre‐assessment.

	TERA (*n* = 16)	Control (*n* = 17)	Test statistic	*p*	Effect size
Mean age in years (SD)	39.25 (12.75)	33.88 (13.23)	*t* (31) = 1.19	.245	*d* = .41
Gender: Women (%)	9 (56%)	16 (94%)	*χ* ^2^ = 6.44	.017	–
PDD (%)	5 (31%)	7 (41%)	*χ* ^2^ = 3.51	.554	–
Current psychotropic medication (%)	4 (25)	7 (41)	*χ* ^2^ = .97	.325	–
Mean No. of therapy sessions (SD)	1.63 (3.58)	.71 (1.44)	*t*(31) = .98	.335	*d* = .34
Mean GERT‐S (SD)	.66 (.09)	.73 (.10)	*t*(31) = −2.01	.053	*d* = .74
Mean PHQ‐9 (SD)	12.94 (5.27)	13.24 (4.29)	*t*(31) = −.18	.860	*d* = .06
IIP‐12 (SD)	21.25 (5.60)	22.94 (7.69)	*t*(31) = −.72	.478	*d* = .25

Abbreviations: GERT‐S, Geneva emotion recognition test‐short version; IIP‐12, Inventory of interpersonal problems; PDD, Persistent depressive disorder; PHQ‐9, Patient health questionnaire‐9.

### Effects of TERA on emotion recognition

There was no significant main effect of group, *F*(1,31) = 1.14, *p* = .293, but a significant main effect of time, *F*(1,31) = 34.90, *p* < .001 (see Table 2). In support of our hypothesis, this was qualified by a significant time × group interaction effect, *F*(1,31) = 4.84, *p* = .035, *d* = .61, indicating that the magnitude of change in ERA differed between groups. Follow‐up paired samples *t*‐tests for each group showed a significant increase in ERA in the TERA group *t*_TERA_(15) = −5.29, *p*_TERA_ < .001, with a large effect size (*d*_TERA_ = 1.32). The increase in ERA in the control group, *t*_Control_(16) = −2.86, *p*_Control_ = .011, was of a medium effect size (*d*_Control_ = .69).

**TABLE 2:** Descriptive statistics and group comparisons on outcome measures.

	Pre‐assessment				Post‐assessment				Test statistic	*p*
	TERA (*n* = 16)		Control (*n* = 17)		TERA (*n* = 16)		Control (*n* = 17)			
	*M*	SD	*M*	SD	*M*	SD	*M*	SD	Group × time	
GERT‐S	.66	.09	.73	.10	.77	.12	.78	.12	*F*(1, 31) = 4.84	*p* = .035
PHQ‐9	12.94	5.27	13.24	4.29	11.68	5.58	11.71	3.67	*F*(1, 31) = .08	*p* = .774
IIP‐12	21.25	5.60	22.94	7.69	19.19	7.05	23.00	7.91	*F*(1, 31) = 3.53	*p* = .070

Abbreviations: GERT‐S, Geneva emotion recognition test‐short version; IIP‐12, Inventory of interpersonal problems; PHQ‐9, Patient health questionnaire‐9; TERA, Training of emotion recognition abilities.

### Effects of TERA on depressive symptoms

There was no significant main effect of group, *F*(1,31) = .01, *p* = .921, but a significant main effect of time, *F*(1,31) = 8.32, *p* = .007, indicating that both groups showed lower PHQ‐9 scores after training. However, there was no significant time × group interaction, *F*(1,31) = .08, *p* = .774, *d* = .06, indicating that the magnitude of change in PHQ‐9 scores was comparable in the two groups (see Table 2).

### Effects of TERA on interpersonal problems

There was no significant main effect of group, *F*(1,31) = 1.29, *p* = .265 or time, *F*(1,31) = 3.15, *p* = .086, and no significant time × group interaction effect, *F*(1,31) = 3.53, *p* = .070, *d* = .31. This indicates that groups did not differ regarding interpersonal problems and that none of the groups experienced significant changes in interpersonal problems (see Table 2).

### Moderator analyses

Neither the association between group assignment and change in ERA (all *p* > .549) nor that between group assignment and change in depression (all *p* > .271) was moderated by age, gender, psychotropic medication, the number of previous psychotherapy sessions in the outpatient clinic in the recent past or length of time between training and post‐assessment. In addition, the level of depression at pre‐assessment did not moderate the association between group assignment and change in ERA (*p* = .284). The difference in the magnitude of ERA change between groups depended to some degree on the length of time between training and post‐assessment, Δ*R* ^2^ = 12.60%, *F*(1, 29) = 5.22, *p* = .029, 95% CI[−.001, .015]. However, this effect was no longer significant after Bonferroni correction for multiple testing.

Furthermore, the association between group assignment and change in interpersonal problems was not moderated by any of the baseline variables (age, gender, number of previous psychotherapy sessions in the outpatient clinic in the recent past, medication, depressive symptoms, time between training and post‐assessment; all *p* > .199).

## DISCUSSION

The present study aimed to examine whether ERA can be enhanced in patients with depression and whether such changes have an impact on interpersonal problems and symptoms of depression. To this end, forty outpatients with depression were randomly allocated to receive a single session of either TERA or a sham training. Participants in both conditions showed improvements in ERA and depressive symptoms but not in interpersonal problems 2 weeks after training. Participants who received TERA showed greater improvements in ERA but not in interpersonal problems or depressive symptoms. This suggests that ERA can be effectively trained in patients with depression, but that enhanced ERA does not necessarily translate into improvements in interpersonal problems and depression.

### Effects of TERA on ERA

Previous research found that ERA can be improved in patients with other mental disorders (Berggren et al., 2018; Penton‐Voak et al., 2021; Preis et al., 2020). The present study is the first to show that ERA can also be trained in patients with depression. Patients who received TERA showed greater improvements in ERA than patients who received sham training. The magnitude of improvement in ERA, as measured by the GERT‐S (11%), is comparable to that seen in previous studies in patients with anorexia nervosa (12% in Preis et al., 2020) and in studies of healthy individuals (Schlegel et al., 2018). The lack of any moderating effects of age, gender, number of previous psychotherapy sessions in the outpatient clinic in the recent past, medication and depressive symptoms at baseline suggests that TERA is effective for a broad range of patients. One previous study also examined the effects of an emotion recognition training in depression (Penton‐Voak et al., 2021). However, this study used an analogue sample of volunteers with elevated symptoms of depression, not a clinical sample of patients with MDD or PDD. Furthermore, although the authors of this study termed their intervention ‘emotion recognition training’, it was rather an interpretation bias modification training that involved visual cues of ambiguous emotional expressions, and participants were trained to interpret an expression as happy rather than sad. This intervention is quite different from one that trains participants to decode various nonverbal cues across a broad range of emotions.

In line with previous research (Bänziger et al., 2012), participants in the control group showed a significantly smaller but still visible ERA improvement. As the stimuli of the GERT‐S were presented both at pre‐ and post‐assessment, this result can possibly be attributed to (small) familiarity and practice effects. This means that repeated exposure to the audiovisual presentation of the emotional stimuli alone (even without any explanation and feedback) may trigger learning processes and thus enhance performance.

### Effects of TERA on depression and interpersonal problems

As Krause et al. (2021) pointed out, the association between reduced ERA and depression might be bidirectional. Neurological changes linked to MDD might interfere with emotional processing, which impairs socio‐emotional functioning which in turn might maintain depression. At the same time, individuals with reduced ERA are considered to be more vulnerable for interpersonal problems and thereby also for depression (Krause et al., 2021). With this in mind, the second aim of the present study was to investigate whether improving ERA causes (short‐term) changes in interpersonal problems and depressive symptoms. If this is the case, this would support the hypothesis that low ERA contributes to the maintenance of depression. As expected, depressive symptoms decreased significantly over time. However, groups did not differ in terms of this improvement. This corresponds to the study by Penton‐Voak et al. (2021) who, however, used a different kind of intervention and no clinical sample. Since no baseline variables moderated the link between group assignment and change in depression, there seem to be no subgroups of patients who benefited from the training in terms of their symptomatology. In terms of interpersonal problems, neither of the two groups showed any significant improvement (despite a small effect size in the expected direction).

However, some issues need to be taken into account when interpreting the findings. The time span of 2 weeks between training and post‐assessment might have been too short for ERA improvements to have an impact on social interactions that could in turn have an effect on depressive symptoms. As the items of the PHQ‐9 refer to the experience of depressive symptoms over the last 2 weeks, enhanced ERA (through TERA) must have had an immediate effect on social interactions to make a measurable difference at post‐assessment. Moreover, it is debatable whether or not patients were able to transfer their enhanced ERA to real‐life interactions. If this transfer fails, patients might not experience positive changes in social interactions, which otherwise might have instigated decreased depressive symptoms. In this regard, it might be important that the study was conducted during a period of repeated social restriction due to the COVID‐19 pandemic, which might have hindered participants to engage and thus to experience change in social interactions.

The fact that all patients, irrespective of group affiliation, showed reduced depressive symptoms is not surprising as some of them began or continued their outpatient treatment. In addition, nonspecific effects like regression to the mean, spontaneous remission and demand effects may have contributed to the improvement in depressive symptoms seen in both groups.

### Limitations

In addition to the limitations already mentioned, the following limitations need to be considered when interpreting the findings. The sample was limited to rather younger female participants. As previous studies suggest a small but significant gender difference in ERA favouring women (Schlegel et al., 2018), future studies may use gender as a stratification factor in randomization to avoid unequal allocation of participant gender to treatment groups. However, in the present study, there was neither a significant gender difference in ERA nor a moderating effect of gender on changes in outcomes.

Furthermore, we have neither control nor information about the conditions that surrounded participants during the tasks. Although participants were instructed to complete the tasks alone in a quiet place, we cannot exclude that participants asked others for help or were distracted to some degree by the surroundings. Despite these limitations tied to online studies, one advantage is that we could reach patients with depression that would not have come to (additional) face‐to‐face meetings (during the pandemic).

It should also be taken into account that the control condition only controlled for participants actively working on a task involving object recognition and differentiation. The type of control group obviously could not control for expectation effects and was not suitable for testing the effects of exactly this type of TERA (vs. possible other types of TERA).

Another limitation is certainly that the intervention consisted of only one session. It is quite possible that effects regarding ERA stabilize with increasing training frequency. Future interventions may encompass methods to motivate and facilitate transfer into real life (e.g. daily diary after training on how attention to nonverbal signals was paid). Furthermore, future studies may examine not only interpersonal problems but also variables such as social support and satisfaction in interpersonal interactions as both outcome and potential mediator. Finally, the follow‐up period may not have been ideal for measuring changes in depression triggered by improvement in ERA. On the one hand, 2 weeks might have been too short for ERA improvements to have an impact on social interactions that could, in turn, have an effect on depressive symptoms. On the other hand, the period might have been too long to assess short‐term effects like those found in patients with anorexia nervosa after receiving TERA (Preis et al., 2020). Therefore, future studies should include multiple follow‐up assessments.

## CONCLUSIONS

The present study provides first evidence that a single session of a computerized online training of ERA can effectively improve ERA in patients with MDD. However, the training improved neither depressive symptoms nor interpersonal problems. Further studies in larger samples using repeated training, different measures of potential action mechanisms and more follow‐up assessments are needed to examine whether and how this kind of intervention can contribute to countable and sustainable improvements in depression.

## AUTHOR CONTRIBUTIONS

**Mira A. Preis:** Formal analysis; investigation; project administration; writing – original draft. **Katja Schlegel:** Methodology; resources; software; writing – review and editing; supervision. **Sophie Rehbein:** Investigation; writing – review and editing. **Katja Lorenz:** Investigation; writing – review and editing. **Timo Brockmeyer:** Conceptualization; methodology; formal analysis; resources; writing – review and editing; supervision; project administration.

## CONFLICT OF INTEREST STATEMENT

The authors declare no potential conflict of interest.

## Data Availability

The dataset generated and analysed in the current study can be obtained in an anonymized format from the corresponding author upon reasonable request.
